# Pairing and recombination features during meiosis in *Cebus paraguayanus *(Primates: Platyrrhini)

**DOI:** 10.1186/1471-2156-10-25

**Published:** 2009-06-05

**Authors:** Raquel Garcia-Cruz, Pedro Robles, Eliana R Steinberg, Nuria Camats, Miguel A Brieño, Montserrat Garcia-Caldés, Marta D Mudry

**Affiliations:** 1 Unitat de Biologia Cellular i Genètica Mèdica, Facultat de Medicina, Universitat Autònoma de Barcelona, Spain; 2 Grupo de Investigación en Biología Evolutiva (GIBE), CONICET, Departamento de Ecología, Genética y Evolución, FCEyN, Universidad de Buenos Aires, Buenos Aires, Argentina; 3 Grupo de Investigación en Biología Evolutiva (GIBE), Departamento de Ecología, Genética y Evolución, FCEyN, UBA, Cdad, Universitaria, Pabellón II, 4to Piso, Lab 46 (1428 EHA), Cdad, Autónoma de Bs As, Buenos Aires, Argentina

## Abstract

**Background:**

Among neotropical Primates, the Cai monkey *Cebus paraguayanus *(CPA) presents long, conserved chromosome syntenies with the human karyotype (HSA) as well as numerous C+ blocks in different chromosome pairs.

In this study, immunofluorescence (IF) against two proteins of the Synaptonemal Complex (SC), namely REC8 and SYCP1, two recombination protein markers (RPA and MLH1), and one protein involved in the pachytene checkpoint machinery (BRCA1) was performed in CPA spermatocytes in order to analyze chromosome meiotic behavior in detail.

**Results:**

Although in the vast majority of pachytene cells all autosomes were paired and synapsed, in a small number of nuclei the heterochromatic C-positive terminal region of bivalent 11 remained unpaired. The analysis of 75 CPA cells at pachytene revealed a mean of 43.22 MLH1 foci per nucleus and 1.07 MLH1 foci in each CPA bivalent 11, always positioned in the region homologous to HSA chromosome 21.

**Conclusion:**

Our results suggest that C blocks undergo delayed pairing and synapsis, although they do not interfere with the general progress of pairing and synapsis.

## Background

During the first meiotic prophase, very specialized events, such as pairing, synapsis and recombination, take place. Pairing of homologous chromosomes refers to the recognition and alignment of chromosomes, while synapsis means the physical connection between them by the formation of a tripartite proteinaceous structure called the Synaptonemal Complex (SC) [1,2]. Recombination is the process by which exchanges between homologous chromosomes occurs, leading to the formation of chiasmata. Many proteins involved in the recombination process have been identified in recent years. Replication Protein A (RPA) is a component of the transitional meiotic nodules [3], while MLH1 is a marker of crossing-over (CO) events [4]. The meiotic prophase progress is regulated by different checkpoints, and among them the pachytene checkpoint recognizes the presence of unpaired chromosomes and leads to their silencing through the recruitment of proteins such as BRCA1 and ATR [5,6].

Primates represent the mammalian model which is closest to humans and the most frequently used as an experimental model, after rodents. Therefore, the study of the meiotic prophase in non-human primates could be useful for the study of conserved features among primate species, such as the synaptic and recombination processes. Furthermore, the study of the meiotic prophase in Primate species could also be useful to understand the pairing-synapsis behavior of non-centromeric heterochromatin.

The Cai monkey *Cebus paraguayanus *(CPA) belongs to the Cebidae, the widest and most diverse family of New World primates [7-9]. The taxonomy of the genus is still under debate. While some authors recognize four species [8], others recognize five [10] or six [7], therefore emphasizing the importance of performing genetic studies as a tool for taxonomic as well as phylogenetic analyses. Cytogenetic studies in neotropical Primates have demonstrated that *Cebus *presents long conserved chromosome syntenies with the human karyotype (HSA) [11,12]. Some of these homeologies are associated in the CPA karyotype with characteristic blocks of non-centromeric heterochromatin. These regions are C-positive (C+) after C-banding. CPA possesses a great amount of constitutive heterochromatin both at terminal and interstitial positions in different chromosome pairs, thus it constitutes an excellent animal model to analyze the behavior of these regions during mitosis and meiosis [13-16]. This non-centromeric heterochromatin has been previously characterized by C-banding, restriction-enzymes banding and Fluorescent *in situ *Hybridization (FISH) techniques in mitotic cells from different sources [17-22].

Previous analyses have also shown that these regions possess different kinds of repeated DNA sequences. Particularly, CPA chromosome 11 contains a terminal heterochromatic block that is polymorphic and takes up to 75% of the total chromosome length. FISH experiments allowed us to identify the presence of the 3/21 synteny in the euchromatic region of CPA chromosome 11, located between the centromere and the terminal heterochromatic block. Cytological and molecular studies showed that the segment homeologous to HSA chromosome 3 is located adjacent to another segment homeologous to HSA chromosome 21 [23,24].

Heterochromatin shows a particular behavior during meiotic pairing and synapsis. In *Drosophila melanogaster*, heterochromatin has been shown to be crucial for chromosome synapsis and segregation (for a review, see [25]). Moreover, the SC in heterochromatic regions shows specific characteristics such as decreased length and increased thickness [26].

Meiotic studies in neotropical Primate species are very scarce, and all of them have used classical cytogenetic approaches [27-35]. Recent studies support the view that most recombination events occur at highly localized hot spots, whereas the bulk of DNA is "cold" (for a review, see [36]). In mammals, levels of recombination vary among species, among chromosomes within species, and among regions within chromosomes. This heterogeneity may affect levels of diversity, efficiency of selection, and genome composition, as well as perhaps having practical consequences for the genetic mapping of traits [37]. To our knowledge no study on crossover frequency has been performed to-date in non-human Primates.

Taking into account that the CPA karyotype presents large blocks of non-centromeric heterochromatin, our aim was to analyze whether the presence of these blocks could have any effect on the meiotic processes of pairing, synapsis or recombination. Immunofluorescence (IF) against different meiotic proteins (REC8, RPA, MLH1, SCYP1, BRCA1, RNA Polymerase II) and fluorescence *in situ *hybridization (FISH) with human whole-chromosome paint probes (WCP) for HSA chromosomes X and 21 were applied on CPA testicular material for the first time, in order to analyze the meiotic behavior of CPA chromosomes, particularly CPA11, which presents a large heterochromatic terminal block.

## Results

### Localization of heterochromatin blocks in somatic and meiotic chromosomes

**Figure 1:**
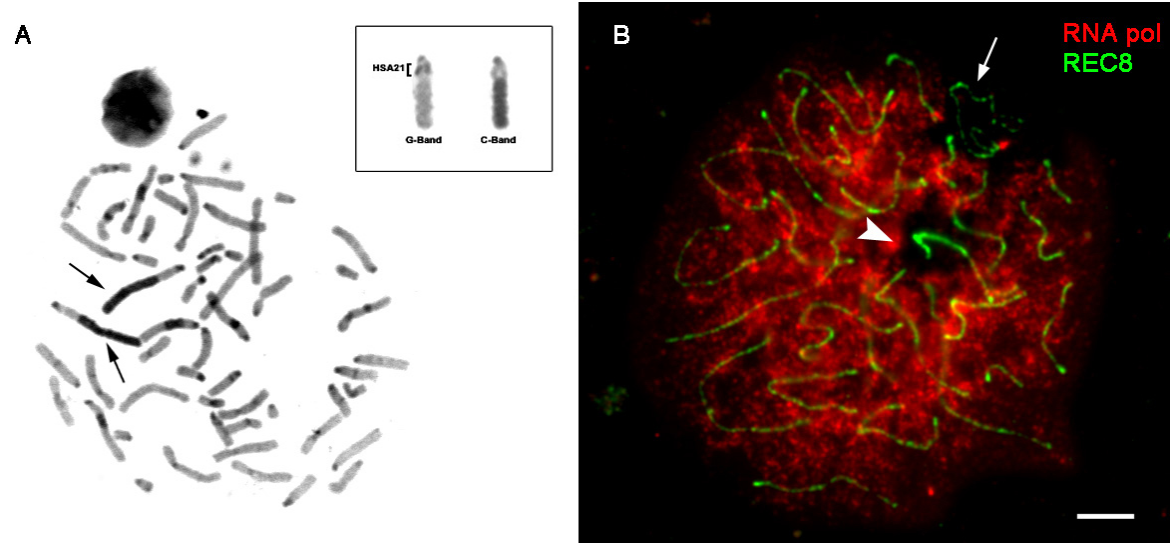
**Identification of the heterochromatic block in CPA 11 on somatic and meiotic cells**. (A) C-band staining of a somatic metaphase. CPA 11 chromosomes are clearly identified by black arrows indicating the presence of large terminal heterochromatic blocks. The inset shows one CPA 11 homolog with sequential G-C- banding. Homology with HSA 21 chromosome is marked through brackets. (B) Pachytene spermatocyte showing diminished RNA polymerase staining on two large domains: the XY body (arrow) and the heterochromatin block of CPA 11 (arrowhead). Bar represents 10μm.

CPA chromosome 11 presents a terminal heterochromatin block which is highly polymorphic, as previously reported [13-22]. C- and G-band analyses of somatic metaphases revealed that the terminal heterochromatic block in CPA chromosome 11 was very long, with a length equivalent to more than half of the total length of the chromosome (Fig. 1A).

When CPA spermatocyte preparations were stained with an antibody against RNA polymerase II, two large domains did not show any signal at the pachytene stage (Fig. 1B). One of the domains corresponded to the XY body, which is known to be transcriptionally silenced during mammalian meiosis. The other domain without signal corresponded to the terminal segment of CPA chromosome 11q, as confirmed by posterior FISH analysis with a specific probe for HSA 21. Other small domains without the RNA polymerase II signal were centromeres and other smaller heterochromatic regions characteristic of the CPA karyotype previously described [17-22].

### Chromosome pairing and synapsis in heterochromatic regions

**Figure 2:**
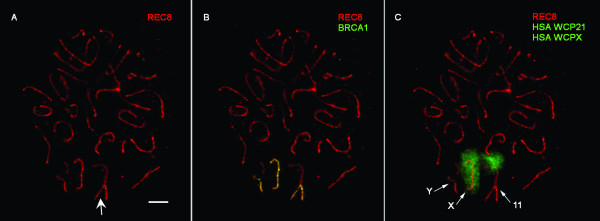
**Pachytene CPA spermatocyte**. (A, B) One SC shows terminal asynapsis (arrow), confirmed by BRCA1 staining. Two other elements are stained by BRCA1, indicative of their asynapsed status. (C) Posterior FISH analysis revealed that unsynapsed chromosomes correspond to CPA chromosomes 11 and X. The third unsynapsed element should be the Y chromosome. Bar represents 10μm.

CPA meiotic preparations were stained with antibodies against BRCA1 and REC8 in order to visualize the pairing of axial elements and the regions that remained unpaired or showed delayed pairing during the meiotic prophase. A low number of nuclei (n = 12/200) was found where all autosomes were completely paired except for one bivalent in which a terminal portion remained unpaired (Fig. 2A). The unpaired axial elements were highlighted with BRCA1 antibody (Fig. 2B). When these preparations were later hybridized with the HSA WCP21 probe, we found that the CPA chromosome pair with the unpaired region was CPA bivalent 11, which has the 3/21 synteny. Considering that CPA 11 has homeology with HSA 21 in the region adjacent to the terminal heterochromatic block, it could be ascertained that the terminal unpaired portion corresponded to the heterochromatin of CPA 11q (Fig. 2C). In six of the above-mentioned CPA nuclei (6/12), the X and Y chromosomes were unconnected showing asynapsis of their pseudoautosomal region (PAR), and they also showed BRCA1-positive signal (Fig. 2B and 2C), four nuclei (4/12) showed a normal XY body, and in the remaining two nuclei (2/12) the synaptic status of the X and Y chromosomes could not be clearly determined.

**Figure 3:**
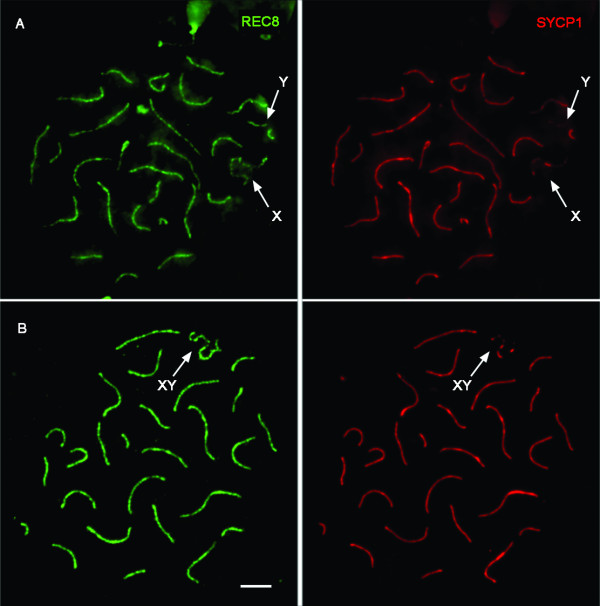
**Two pachytene CPA spermatocytes**. (A) The X and Y chromosomes remain unpaired. SYCP1 staining could be seen on the unpaired X and Y axes. (B) The XY body is formed. More than one SYCP1-positive area could be seen on the unpaired X axis. Bar represents 10μm

Despite the finding of these few nuclei with unpaired heterochromatin in CPA bivalent 11, spermatocyte preparations generally showed the presence of pachytene nuclei with all autosomes completely paired, as revealed by the REC8 signals. It is noteworthy that the REC8 signal in the CPA heterochromatin block often appeared thickened in pachytene spermatocytes (not shown). The question arose as to whether the heterochromatic block really synapsed, although delayed, and if a complete SC between the two homologs was formed. For this purpose, spermatocyte preparations were stained with antibodies against REC8 and SYCP1, a protein of the central element of the SC. A complete co-localization was observed between REC8 and SYCP1 in all CPA autosomes at late-pachytene (Fig. 3), meaning that the large CPA heterochromatic block not only did pair but also synapse along all of its length in spite of the different thickness of its SC.

### Synaptonemal complex formation and XY analysis

CPA meiotic preparations stained with REC8 and SYCP1 antibodies were analyzed to study the pairing-synapsis process. Cohesin cores appeared at pre-leptotene, as revealed by the diffuse REC8 signal. At leptotene, thin cohesin threads were observed at the axial elements. At zygotene, SYCP1 appeared for the first time at the areas of synapsis. Initiation of autosome pairing in CPA spermatocytes was mainly subtelomeric, as revealed by REC8 and SYCP1 immunostaining (not shown). Sex chromosomes XY were the last to initiate synapsis, as revealed by the finding of early-pachytene nuclei with all autosomes (including chromosome 11) synapsed along all of their lengths but with still unconnected X and Y chromosomes (Fig. 3A). Strikingly, in spite of their un-synapsed status, the X and Y chromosomes showed SYCP1-positive regions (Fig. 3A). Once the XY body formed, CPA XY morphology resembled the one observed in human spermatocytes. However, when staining with REC8 and SYCP1, we observed the presence of more than one SYCP1-positive region in the sex body (Fig. 3B).

### MLH1 analysis

IF against MLH1 and REC8 was performed to identify cross-over sites. The analysis of 75 pachytene cells revealed a number of MLH1 foci ranging from 33 to 51, with a mean value of 43.22 ± 5.38 per pachytene nucleus (Coefficient of variation, CV = 12.45%).

**Figure 4:**
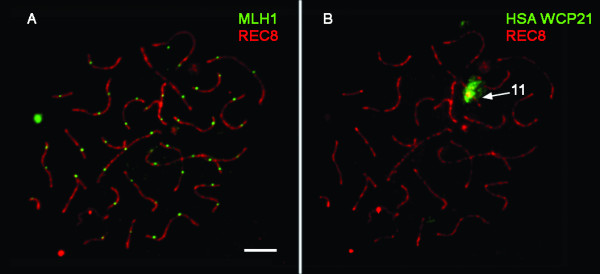
(A) Pachytene CPA spermatocyte stained for the mismatch-repair protein MLH1, which indicates sites of crossing-over. (B) Posterior FISH analysis reveals the identity of SC11. Bar represents 10μm.

MLH1 on CPA chromosome 11 was also analyzed in 83 pachytene nuclei after hybridization of IF-stained preparations with a HSA WCP21 probe. In 92.77% of the cells (n = 77) a single MLH1 focus was detected (Fig. 4A and 4B), while in the remaining 7.23% (n = 6) two MLH1 foci were present. These data represent a mean value of 1.07 MLH1 foci per CPA bivalent 11.

The relative position of MLH1 foci on CPA bivalent 11 was analyzed in 25 of these nuclei. MLH1 always appeared within the segment homeologous to HSA chromosome 21, as revealed by the HSA WCP21 painting probe (Fig. 4A and 4B). In brief, MLH1 foci tended to appear in an interstitial position next to the chromosome centromere. This location was maintained when two MLH1 foci were present, but the second MLH1 tended to appear interstitially, near the heterochromatic block.

### RPA analysis

**Figure 5:**
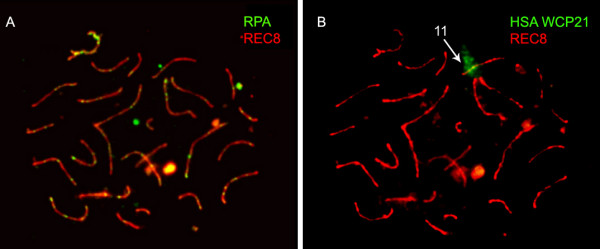
**(A) Pachytene CPA spermatocyte immunostained against REC8 (red) and RPA (green)**. (B) Posterior FISH analysis with a HSA WCP21 probe.

CPA spermatocyte preparations were stained with antibodies against RPA and REC8 (Fig. 5A) followed by hybridization with a HSA WCP21 probe (Fig. 5B) for the analysis of the evolution of transitional recombination nodules.

The RPA signal was observed from the zygotene stage until pachytene. Considering that RPA foci values decrease as pachytene progresses in HSA meiocytes [38,39], we divided pachytene-stage spermatocytes into two sub-stages according to SC condensation and in order to confirm if that was also true for CPA spermatocytes.

The analysis of 30 CPA early-pachytene spermatocytes revealed that the mean RPA foci number was 86.23 per CPA nucleus, with a range from 21 to 135 foci. The 15 CPA late-pachytene nuclei analyzed revealed a mean RPA foci number of 14.23, with a range from 0 to 53 foci, confirming that the RPA foci number also decreases as pachytene progresses in CPA spermatocytes.

RPA distribution on CPA bivalent 11 was analyzed in 15 pachytene nuclei. It was found that RPA was distributed throughout CPA chromosome 11 at early-pachytene, while late- pachytene nuclei showed RPA only in the euchromatic region, suggesting a faster loss of RPA from the non-centromeric large 11q heterochromatic block.

## Discussion

### Heterochromatin behavior

The analysis of pairing-synapsis behavior of the large heterochromatic block in CPA chromosome 11 has revealed that this is the last autosome to pair and synapse completely during the CPA meiotic prophase. The observation that large heterochromatin blocks are the last to synapse has also been described in other organisms [40,41] as well as in human spermatocytes [42]. In *Drosophila melanogaster*, the SC formed between heterochromatic regions shows specific characteristics such as decreased length and increased thickness [26]. This last feature has also been observed by immunofluorescence in the course of our study in the heterochromatic block of CPA spermatocytes, probably due to the different degree of condensation of the chromatin in the heterochromatic regions.

The need of euchromatin for homologue recognition and pairing initiation has already been described. However, the presence of numerous pachytene nuclei with complete synapsis in all autosomes (including CPA11) seems to indicate that initial asynapsis of heterochromatin is rapidly solved and that the large heterochromatin blocks do not only pair but also synapse completely. This has been demonstrated by the presence of fully formed SC as revealed by the presence of the central element protein SYCP1. In addition, the presence of large CPA heterochromatin blocks does not disturb pairing and synapsis in adjacent euchromatic regions of the same CPA chromosome since synapsis of the proximal end of CPA chromosome 11 seems to occur simultaneously with the rest of the CPA autosomes.

### Synaptonemal complex formation and XY analysis

The fact that the initiation of synapsis mainly occurs at telomeres matches the situation described in other mammalian species' spermatocytes and is consistent with findings that point to telomeres as promoters of homologue encounter and pairing initiation at early stages of meiotic prophase through the acquisition of the bouquet topology [43].

In most mammalian species, XY synapsis is restricted to a small region of homology between the X and Y chromosomes called the pseudo-autosomal region (PAR). Previous studies in CPA spermatocytes by silver nitrate staining have revealed an XY morphology similar to the HSA XY pair, considering that CPA presents a human-like sexual determination system clearly identified in both mitotic and meiotic analyses [22-24,27-32]. The use of immunofluorescent techniques in the present study confirms the findings reported by silver nitrate staining. However, the use of immunofluorescence allows for the visualization of specific proteins, which in turn has permitted the visualization of more than one SYCP1 region in the XY pair. This feature has not been observed in HSA spermatocytes (R. García and M. García, unpublished data), although it has been described before in other organisms [44]. We do not think that this corresponds to more than one pairing site between the X and Y chromosomes mainly because of the observation of still unpaired X and Y chromosomes showing this same feature and because of the morphology of the XY pair. Therefore, we suggest that those SYCP1 areas could correspond to areas of self-synapsis between repetitive sequences along the X and Y unpaired axes. This mechanism would allow those sequences to escape from silencing by evading BRCA1-ATR recognition [5]. Nevertheless, the cellular significance that this specific behavior could have remains unclear. Recently, it was shown that SYCP1^-/- ^mice present testicular abnormalities. The analyzed spermatocytes exhibited impaired synapsis and XY body formation, suggesting that this protein may have a role in SC assembly, meiotic recombination and XY body formation [45]. And, on the other hand, double-strand breaks (DSBs) occur at the non-PAR axes of X and Y chromosomes in the male meiotic prophase [46], contrary to the prevalent view that the formation of the XY body protects the non-PAR regions from DSBs.

Alternatively, the SYCP1 regions could correspond to protein aggregates along the axes, an element that was already observed by silver nitrate staining [32]. Hence, more studies should be performed to test these hypotheses.

### Recombination features

The analysis of RPA and MLH1 proteins in CPA spermatocytes has revealed that the general landmarks of the meiotic recombination process in CPA species are similar to the human ones. RPA and MLH1 values at pachytene are similar between CPA and HSA spermatocytes [47,38], and this is also the case for MLH1 CV between both species (data calculated from [47-53]). However, the number of bivalents differs substantially between the two species. The CPA autosomal karyotype is formed by 10 submetacentric plus 16 acrocentric pairs. Therefore, taking into account that submetacentric bivalents should bear at least one CO per arm, and acrocentric bivalents and the XY pair should bear at least one CO event to ensure homologous attachment until metaphase I, it can be assumed that CPA spermatocytes should bear at least 38 COs. The mean number of MLH1 foci in CPA spermatocytes was 43.22, and no bivalents without MLH1 have been observed during the analysis. Thus, it could be assumed that, despite the presence of several heterochromatic blocks, recombination in CPA spermatocytes is an efficient process, where an appropriate amount of COs are formed, leading to a correct alignment of all bivalents at the metaphase I plate and segregation at anaphase I.

The analysis of MLH1 distribution on CPA chromosome 11 has shown that, as was expected, the heterochromatic region of the chromosome did not bear any CO event. MLH1 foci were always located in the region homeologous to HSA chromosome 21 while no COs were found in the region homeologous to HSA chromosome 3. This is most probably due to the fact that the region homeologous to HSA chromosome 3 in CPA chromosome 11 is very small and close to the centromere. It is known that pericentromeric regions show repression for CO establishment. The mean number of CO events in the region homeologous to HSA chromosome 21 was very similar to the one reported for the male HSA chromosome 21 (1.07 *versus *0.97) [47].

## Conclusion

Heterochromatin, in its modern view, represents a specific functional state of chromosome regions or whole chromosomes, a specific kind of chromatin packaging required for transcriptional repression/expression. The effect of heterochromatin on pairing and recombination, both of autosomes and gonosomes, has been studied in different organisms [40,41,54,55] (for reviews see [1,40]). Our contribution constitutes the first analysis using immunofluorescence against meiosis-related proteins performed in non-human Primates. The long distal heterochromatin block in CPA chromosome 11 undergoes delayed synapsis even though complete synapsis is finally achieved. In addition, the presence of the large heterochromatin block in chromosome 11 and the interstitial heterochromatin block on other chromosomes do not interfere in the pairing-synapsis process in the CPA spermatocyte. The sexual chromosomes are the last to synapse in CPA spermatocytes forming an XY body which resembles the one in human spermatocytes. Meiotic recombination in CPA chromosome 11 is restricted to the region homologous to HSA chromosome 21, while no recombination event occurred in the region homologous to HSA chromosome 3 or in the heterochromatin block. More studies would be of great interest to clarify the dynamics of meiotic processes and particularly the role of heterochromatin in considering that, in Primates, different genomic strategies may be involved.

## Methods

### Biological material

This study is based on the analysis of mitotic and meiotic cells of one adult specimen of *Cebus paraguayanus*. The specimen was kept in captivity at the Corrientes Biological Station (EBCo), Corrientes Province, Argentina.

A testicular biopsy was obtained, following the protocols of the Guide for Care and Use of Experimental Animals as promulgated by the Canadian Council of Animal Care and the American Society of Primatologists (ASP) Principles for the Ethical Treatment of non-human Primates.

The testicular biopsy was taken by the Veterinarian Alexis Burna, who administered the dose of anesthetics (Zelazol, Ford Dodge) according to the weight of the animal (4.330 kg). The biopsy was taken to GIBE, FCEyN, UBA, Buenos Aires, and kept in liquid nitrogen at -80°C until use.

The peripheral blood sample was collected by the Veterinarian Gustavo Solís with previously heparinized disposable syringes in order to perform cell-suspension cultures.

All research reported in this manuscript has followed the appropriate national and institutional guidelines for the legal acquisition and use of laboratory animals and authorized study of wild animals. The work which took place in Argentina was done in accordance with Argentinian laws.

### Mitotic chromosome preparations

The blood sample was separated into two. Peripheral-blood lymphocyte cultures were performed in duplicate for 72 hours at 37°C at GIBE and at the Unitat de Biologia Cellular i Genètica Mèdica, following standard techniques [[56], modified]. Air-dried metaphase spreads were treated for G-banding [[57] modified], and C-banding [58] techniques. At least 50 metaphases were analyzed to confirm the species chromosome number, and 10 G-banded and 10 C-banded complete metaphases (2n = 54) were photographed and karyograms were constructed following the chromosome nomenclature of Ponsà et al. [59].

### Meiotic preparations

Samples were processed following the protocol described by Roig et al. [60] with minor modifications in order to obtain spermatocyte spreads for immunofluorescence. Briefly, a piece of the testicular biopsy was carefully minced on a slide; 80 μl of 1% Lipsol was added on top of the slide. After 8 minutes, 90 μl of a fixative solution containing 1% paraformaldehyde and 0.15% Triton X-100, pH = 9.2, was carefully added, and the slide was kept in a humid chamber. After 2 hours, the slide was washed in 1% photo-flo solution and further processed for immunofluorescence, or frozen at -80°C until use.

### Inmmunofluorescence (IF)

Immuno-staining of meiocytes was performed using the following primary antibodies: mouse and rabbit polyclonal sera against cohesin REC8 (both were kind gifts from J.L. Barbero), rabbit poyclonal serum against the central element protein SYCP1 [61] (kind gift from C. Heyting), rabbit polyclonal antibody against BRCA1 (Abcam), mouse monoclonal antibody against the phosphorylated form of RNA Polymerase II (Covance), mouse monoclonal antibody against MLH1 (Pharmingen) and mouse monoclonal antibody against RPA (Calbiochem). Fluorochrome-conjugated secondary antibodies were used for detection (all from Jackson ImmunoResearch Laboratories).

Antibodies were diluted in PBTG (0.2% BSA, 0.05% Tween, 0.2% gelatin in PBS). Primary antibodies were incubated overnight at 4°C or at room temperature in a humid chamber. Secondary antibodies were incubated for 1 h at 37°C in a humid chamber. After washing away the excess of secondary antibodies, DNA was counterstained with anti-fade solution (Vector Laboratories) containing 0.1 μg/ml DAPI (4',6'-diamidino-2-phenylindole; Sigma).

### FISH on immuno-stained preparations

IF-stained preparations were hybridized with human whole-chromosome painting probes (WCP) for HSA chromosomes X and 21 (both from Cambio). A standard hybridization protocol including long hybridization and low stringency washes was used. Briefly, a first denaturation of the slides was performed in 70% formamide in a 2 × SCC solution for 5 minutes at 70°C. The slides were washed in water, and a treatment with NaSCN 1 M for 3 h at 65°C was applied. A second denaturation step was then performed as stated above. Dehydration of the slides was carried out before applying the denatured probe. Hybridization was performed at 37°C in a humid chamber for 72 hours. Post-hybridization washes were performed in 4 × SCC.

### Microscopy and image analysis

Somatic chromosome preparations were observed using an Olympus BX50 microscope. Images were captured and processed by Cytovision software. Meiotic preparations were observed using an Olympus BX70 fluorescence microscope. Images were captured and processed by Smart Capture software (Vysis) and further processed using Adobe Photoshop to match the fluorescent intensity seen through the microscope.

Micromeasure 3.3 software (available at ) was used for analysis of synaptonemal complexes length.

## Authors' contributions

RGC, PR, NC, MAB, MDM carried out the inmmunofluorescence and the FISH assays. RGC, PR, NC, MAB analyzed the IF and FISH results and contributed to the writing of the paper. ERS organized the collection of the blood and testicular biopsy samples and contributed to the writing of the manuscript. MDM and MGC conceived the study, participated in the development of techniques, coordinated the study and contributed to the writing of the manuscript. MGC and MDM are the mentors and this work was designed by them. All authors read and approved the final manuscript.
